# Autologous stem cell transplantation in HIV-related lymphoma in the rituximab era – a feasibility study in a monocentric cohort

**DOI:** 10.7448/IAS.17.4.19648

**Published:** 2014-11-02

**Authors:** Imke Wieters, Johannes Atta, Gerrit Kann, Junaid Owasil, Siri Goepel, Annette Haberl, Christoph Stephan, Timo Wolf

**Affiliations:** 1 Department of Infectious Diseases, HIV Center, J. W. Goethe University Hospital, Frankfurt, Germany; 2 Haematology and Oncology, J. W. Goethe University Hospital, Frankfurt, Germany

## Abstract

**Introduction:**

Since the introduction of highly active antiretroviral therapy (HAART) [1] and later on the availability of anti-CD20 monoclonal antibody treatment [2], the therapeutic options as well as the prognosis of AIDS related lymphoma (ARL) have been improved. There is however no uniform agreement on how to treat patients who do not achieve a partial remission, who experience a relapse or who have very aggressive subtypes. Autologous hematopoietic stem cell transplantation (ASCT) has become an option for those patients. We retrospectively examined ARL patients to elucidate the feasibility of high-dose chemotherapy and autologous stem cell transplantation.

**Patients and methods:**

Data of seven male and one female HIV+ patients with ARL was collected and informed consent was obtained. Age, HIV disease characteristics (CD4 count, HIV-RNA-PCR, ART) and transplantation-related details (histopathology, myeloablative therapy, neutrophil engraftment and NCI-CTCAE during/after transplantation as well as follow up and survival) were obtained from the patients’ medical records.

**Results:**

Eight patients were treated with the intent of ASCT. The median age was at 64 years. Four patients had experienced prior AIDS. The median CD4 NADIR was at 157/µl, the median CD4 count at diagnosis of lymphoma at 81/µl. Five patients were receiving combination antiretroviral therapy (cART) at the time of lymphoma diagnosis, four of which had achieved a viral load of less than 50/µl. Two patients have died, due to (Nr. 8) a transplant-related complication (non-infectious leukoencephalophathy). The other patient died of an unknown reason (351 days after transplantation). The median survival is at 345 days to date. The time until engraftment was well at 11 days. Grade 3/4 haematological toxicity was present in all patients. Five out of three patients developed infectious complications, but there were no infection-related deaths. One patients (Nr. 4) developed a Kaposi Sarcoma reactivation that necessitated further chemotherapy.

**Conclusions:**

ASCT is feasible in high risk ARL with good engraftment. Toxicity was substantial and studies are needed to define which patients have an unduly high risk of toxicity.

**Table 1:** Baseline characteristics

Gender	Age	Lymphoma	Ann Arbor	Prior AIDS	CD 4 at HIV primary diagnosis	CD4 at lymphoma	Viral load at lymphoma	ART at lymphoma
M	50	DLCL	II B	Kaposi	unknown	134	<20	TDF/FTC/EFV
M	66	DLCL (first relapse)	IV BE	no	753	2	8700000	none
M	30	Plasmablastic Lymphoma	IV BE	Kaposi	165	150	<20	FTC/TDF/RGV
M	36	PCNSL	n/a	Kaposi	unknown	96	817000	FTC/TDF/RGV/DRV/RTV
M	59	DLCL	IV BE	no	171	66	36	FTC/TDF/ATV/RTV
M	36	Plasmablastic Lymphoma	IV BE	no	13	13	265000	non
M	41	DLCL (first relapse)	II	Tuberculosis	154	883	<20	ETV/MVC/DRV/RTV
F	57	PCNSL	n/a	no	3	3	1990000	none

M=Male; F=Female; diffuse large cell lymphoma=DLCL; primary central nervous system lymphoma (PCNSL).
